# An Overview of Recent Standard and Accelerated Molecular Dynamics Simulations of Helium Behavior in Tungsten

**DOI:** 10.3390/ma12162500

**Published:** 2019-08-07

**Authors:** Luis Sandoval, Danny Perez, Blas P. Uberuaga, Arthur F. Voter

**Affiliations:** 1 Theoretical Division T-1, Los Alamos National Laboratory, Los Alamos, NM 87545, USA; 2 Materials Science and Technology Division MST-8, Los Alamos National Laboratory, Los Alamos, NM 87545, USA

**Keywords:** helium bubbles, tungsten, nucleation and growth

## Abstract

One of the most critical challenges for the successful adoption of nuclear fusion power corresponds to plasma-facing materials. Due to its favorable properties in this context (low sputtering yield, high thermal conductivity, high melting point, among others), tungsten is a leading candidate material. Nevertheless, tungsten is affected by the plasma and fusion byproducts. Irradiation by helium nuclei, in particular, strongly modifies the surface structure by a synergy of processes, whose origin is the nucleation and growth of helium bubbles. In this review, we present recent advances in the understanding of helium effects in tungsten from a simulational approach based on accelerated molecular dynamics, which emphasizes the use of realistic parameters, as are expected in experimental and operational fusion power conditions.

## 1. Introduction

Conditions expected in the divertor of the world’s largest tokamak under construction, ITER (“The Way” in Latin, formerly known as International Thermonuclear Experimental Reactor), include the low-energy (≤100 eV) helium nuclei impact on a high-temperature tungsten surface (∼1000 K) [1]. No tungsten defects are created upon impact because the maximum energy transferable from the collision is significantly below the tungsten displacement threshold [2]. The helium atoms diffuse in the tungsten matrix, eventually forming helium clusters with lower diffusion rates. At a critical size, a helium cluster converts into a practically immobile (under Molecular Dynamics (MD) time scales) entity, composed of the helium cluster and a tungsten Frenkel pair [3]. This helium (nano-)bubble is able to collect additional helium clusters, triggering the nucleation of additional Frenkel pairs. As a result, the bubble grows and the tungsten interstitials form a dislocation line pinned to the bubble, which eventually detaches as a loop, effectively displacing tungsten atoms in the matrix [4,5,6].

Recent simulation results, which are the focus of the present review, have shown that the process of the nucleation and growth of He bubbles in W exhibits extremely rich dynamics, involving competing mechanisms defined in an ample interval of time scales, which should be studied by considering advanced atomistic techniques beyond the standard MD approach. In doing so, we mostly focus on work from our own group where so-called Accelerated MD (AMD) techniques have been applied to the problem. This manuscript is therefore not meant to be an exhaustive review of the vast literature in the field, but to summarize our research effort over the last few years. First of all, we briefly review in Section 2 some results concerning the reflection and implantation of He atoms in W, which give information about the initial distribution of diffusing He atoms as a function of the impact energy, angle of incidence, surface orientation and temperature. In Section 3, the kinetics of He clusters is considered, with particular attention on the mobility of small clusters and the phenomenon of trap-mutation, as well as the mobility of small vacancy/helium complexes. The nucleation and growth of He bubbles in W is the focus of Section 4. In Section 5, we present some results from recent studies on the interaction between He clusters and He bubbles. Interaction between He bubbles and defects, specifically grain boundaries, is reviewed in Section 6. Finally, in Section 7, we discuss the advantages of the AMD techniques, as compared to standard MD, which have allowed us to gain fundamental insight into the nucleation and growth of helium bubbles in tungsten.

## 2. Reflection and Implantation of He Atoms

For the sake of completeness, we first discuss the reflection and implantation processes of He atoms in W. In a fusion reactor, the plasma irradiation flux is composed of deuterium, tritium, and helium. Contrary to the other two species, which may form a deposited layer, incoming He atoms are either reflected upon impact or implanted below the W surface. As a first approach to the implantation problem, we can ignore the interaction with deuterium and tritium, in order to focus on the behavior of He atoms.

Borovikov et al. [7] studied this topic via MD simulations, significantly extending the scope of previous computational efforts [8] by considering the effect of temperature in the substrate (300 K, 1000 K, and 1500 K), incidence energy $E_{i}$ (≤100 eV), deposition angles $\theta_{i}$ (0–$75^{\circ }$), and substrate surface orientation ((100), (110), and (310)). The interaction between W atoms was determined by an Ackland–Thetford potential [9], modified at short distances by Juslin and Wirth [10]. He–W interactions were obtained from Juslin and Wirth [10], while the He–He potential corresponded to the one used by Beck [11] modified at short distances by Morishita et al. [12]. Incidentally, these potentials were also used to obtain all results discussed in the following sections. For each set of parameters considered, 1000 non-accumulative He impact simulations were performed, generating the statistics. The main focus was the determination of the reflection coefficient *R* (ratio of reflected He atoms to the total number of impacting He atoms), the energy reflection coefficient $R_{E}$ (ratio of the kinetic energy of the reflected He atom to the incident kinetic energy of the He atom), and the average projected range *L* (implantation depth normal to the surface) for the implanted He atoms.

**Figure 1:**
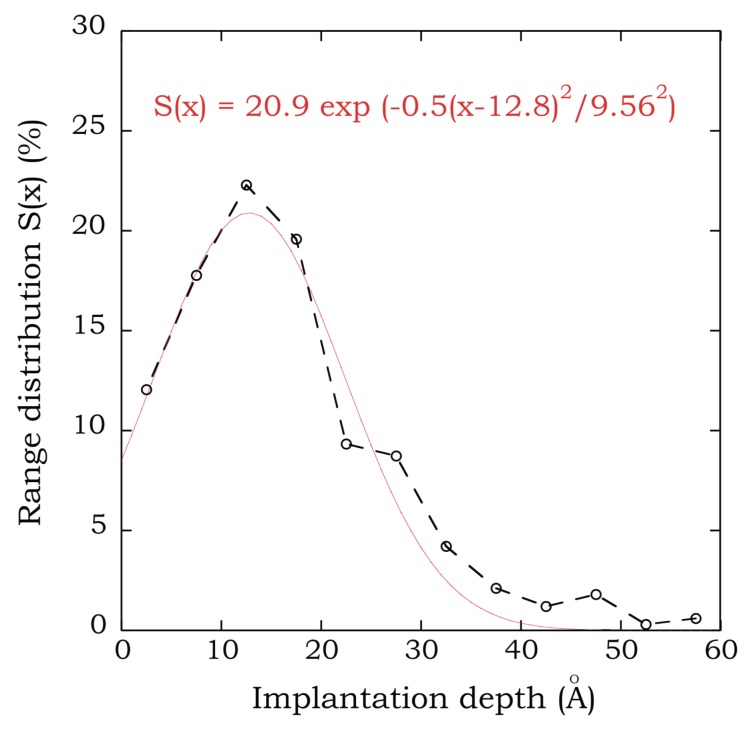
(Color online) He-implantation depth distribution for a (low) impact energy $E_{i}=80$ eV, at a deposition angle $\theta_{i}=0^{\circ }$, on a W (100) surface equilibrated at 1000 K. A fit to a Gaussian distribution truncated at the surface is also shown. Taken from Borovikov et al. [7].

Given a substrate temperature and orientation and a deposition angle $\theta_{i}\leq 75^{\circ }$, Borovikov et al. [7] showed that an increase of impact energy ($E_{i}>10$ eV), in general, implies a decrease of *R* and $R_{E}$ and an increase of *L*. For $\theta_{i}=75^{\circ }$, all the atoms were reflected. Low impact energies ($E_{i}\leq 10$ eV), even for small deposition angles, resulted in a reflection coefficient equal to one. They also observed that the dependence of particle/energy reflection coefficients with the incidence angle was non-monotonic, with a minimum at specific angles (e.g., $\theta_{i}∼$ 30–45${}^{\circ }$ for a surface orientation of (100)), a behavior attributed to channeling effects. At low temperatures (∼300 K), *L* increases substantially at $\theta_{i}=0^{\circ }$ due to the high probability of channeling events [13]. If channeling is absent, the implantation depth distributions can be well described by a Gaussian distribution [13], which, at low impact energies, is truncated at the surface, as shown in Figure 1. More recent works have extended these results by considering additional surface orientations [14], the effects of pre-existing bubbles in W surfaces [15], and curvature effects in W nanoparticles [16].

## 3. Kinetics of He Clusters

Once individual He atoms are implanted into the W matrix, their evolution is dictated by diffusion and clustering. In this section, we highlight some recent results concerning the evolution of interstitial He.

### 3.1. Mobility of Small He Clusters and Trap Mutation

As a He atom diffuses through the lattice, it either encounters traps (point defects, dislocations, etc.) or other He atoms. Clustering is favored because of the binding between He atoms generated by the elastic interactions caused by the repulsion between the metal atoms and He [17]. At a certain size, a growing He cluster can force the emission of W interstitials (crowdions), nucleating vacancies (V) that accommodate the He cluster. This process is known as trap mutation [18] or self-trapping [3].

Using conventional MD, Temperature-Accelerated Dynamics (TAD) [19], Statistical Temperature (STMD) [20], and multicanonical MD [21,22], Perez et al. [23] determined important thermodynamic and kinetic parameters describing the diffusion and transformation of small He clusters in W. They specifically investigated the behavior of He${}_{N}$ clusters with *N* ranging from 2–7 He atoms, in an interval of temperatures relevant to nuclear fusion applications centered around 1000 K.

**Figure 2:**
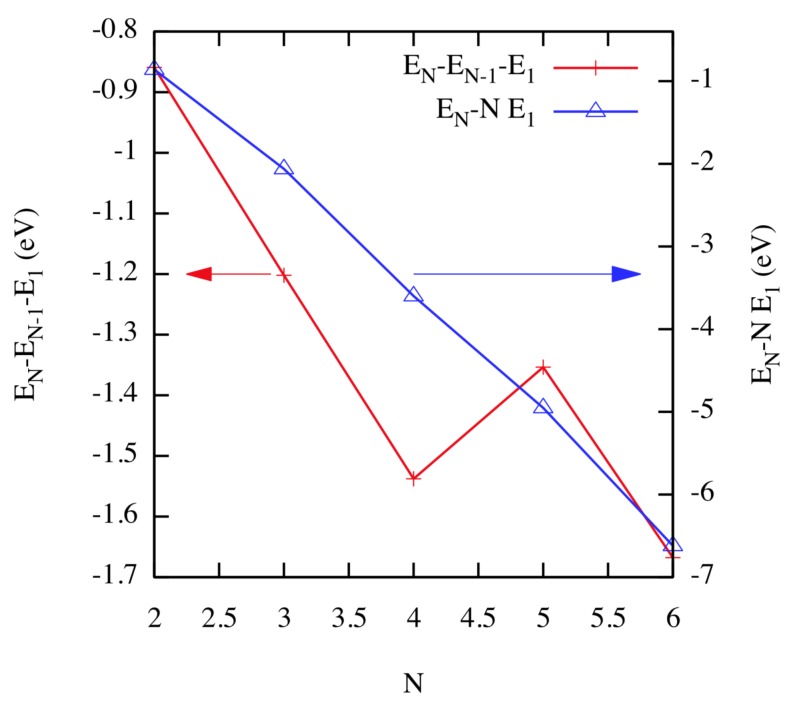
(Color online) Energy change after removing single atoms from a He cluster of size *N* ($E_{N}-E_{N-1}-E_{1}$; red, left axis) and after complete fragmentation into *N* single He atoms ($E_{N}-NE_{1}$; blue, right axis). These values correspond to T=0 K. Taken from Perez et al. [23].

In order to find the most important low-temperature diffusion pathways, Perez et al. [23] performed TAD with low and high target temperatures of 300 K and 600 K, respectively. For a given cluster with size *N*, their simulations started with the lowest energy structure found for the cluster with size N−1 plus one additional He interstitial. Once the two species encountered one another, forming a He cluster with size *N*, its diffusion was simulated. For each cluster, the lowest energy diffusion pathway, as well as the lowest energy structure was identified. With the exception of the case corresponding to N=5, the ground state structure for all the clusters was found to be composed of He atoms located in tetrahedral interstices. For N=5, the fifth He atom occupies an octahedral interstice; note that there are 24 tetrahedral and 18 octahedral positions within the BCC unit cell. As shown in Figure 2, the strength of the binding of the He cluster increases with the cluster size. If we consider the change of energy after removing a He atom from the clusters, the case N=5 corresponds to a local maximum. Density Functional Theory (DFT) calculations [24] show a similar behavior, but with higher values, approximately 30%, as compared to the classical potential used in this work.

**Figure 3:**
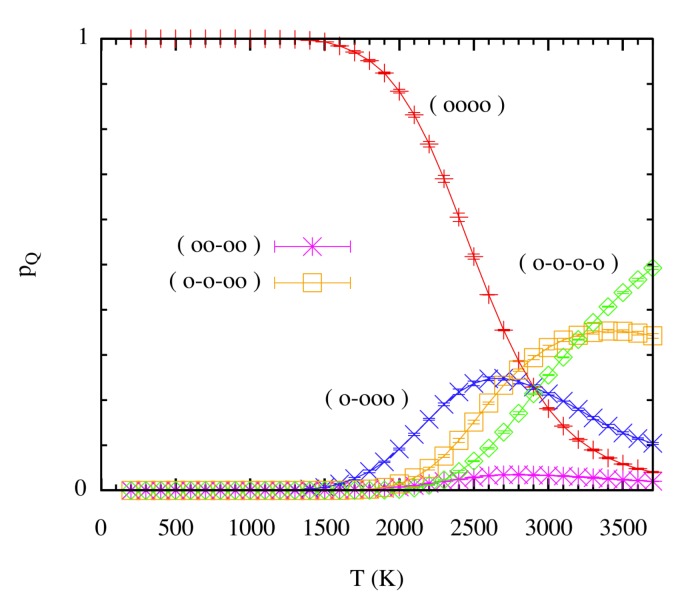
(Color online) Probability of finding a certain cluster distribution $p_{Q}\left(T\right)$ as a function of temperature for the case involving four He atoms (N=4). Notation: (o–o–o–o) = four single atoms; (o–o–oo) = two single atoms plus a two-atom cluster; (oo–oo) = two two-atom clusters; (o–ooo) = one single atoms plus a three-atom cluster; (oooo) = one four-atom cluster. Taken from Perez et al. [23].

Using STMD and multicanonical simulations, Perez et al. [23] obtained finite-temperature effects on the stability of the clusters, specifically the canonical distributions of cluster compositions for all temperatures, which allowed determining the probabilities $p_{Q}\left(T\right)$ of finding certain cluster compositions *Q*. As an example, the case corresponding to N=4 is shown in Figure 3. For temperatures T≤2500 K, the most notable cluster configuration is a cluster containing all four He atoms. With significant probability, single He atoms start to be observed at temperatures above 1500 K. On the other hand, the equilibrium probability of single He atoms becomes vanishingly small at low temperatures. Furthermore, using free energy data, Perez et al. [23] showed that the temperature at which He atoms separate from the clusters, or at which clusters are completely fragmented, grows with *N*, in accordance with the results obtained at T=0 K. Figure 3 also provides a picture of the most probable dissociation scenario as a function of temperature. For instance, approximately between 1500 K and 2700 K, the four-atom cluster most likely transforms to a three-atom cluster plus a dissociated He atom, although the additional dissociation of He atoms starts to be more significant above 2000 K, probably involving intermediate steps. Additional insight is gained when we consider the volume-independent free energy of the clusters, which provides transition points for a given transformation.

**Figure 4:**
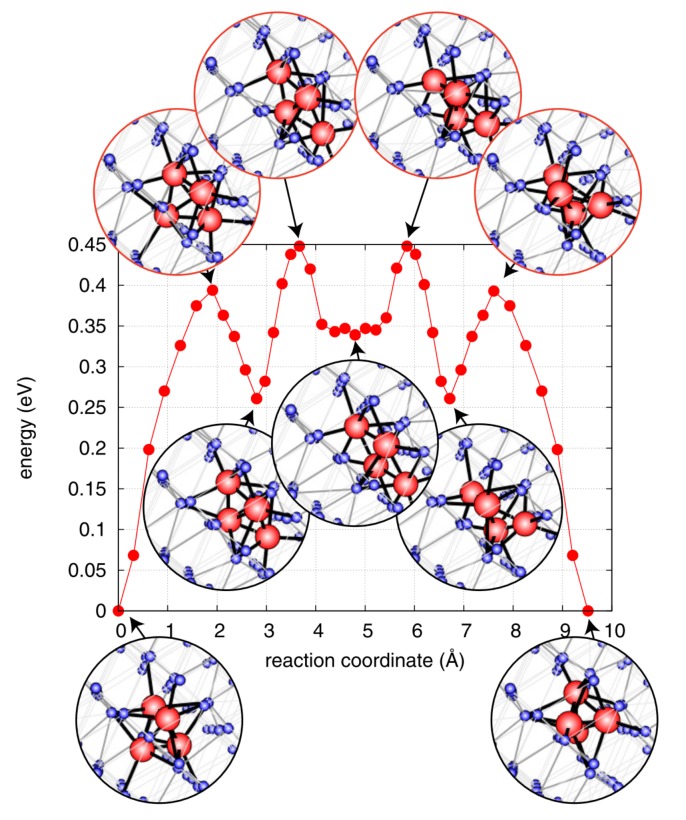
(Color online) Minimum Energy Path (MEP) for a He cluster containing four atoms. Small (blue) and large (red) spheres correspond to W and He, respectively. Taken from Perez et al. [23].

Available transition pathways are characterized via the Nudged Elastic Band (NEB) method [25], which is an inherent step in TAD. This analysis was performed by Perez et al. [23] for He cluster sizes ranging from N=1–N=6. For instance, for N=1, the diffusion pathway goes from a tetrahedral interstice (lowest energy), through an octahedral interstice (saddle point), to another tetrahedral interstice position. The corresponding energy barrier was 0.15 eV. Note that this value is almost one order of magnitude higher than the one obtained via ab initio calculations [26], a result attributed to the limitations of the interatomic potential. The diffusion pathways become more complex (additional intermediate minima) as the He cluster size increases. As an example, Figure 4 shows the lowest energy migration pathway for N=4.

**Figure 5:**
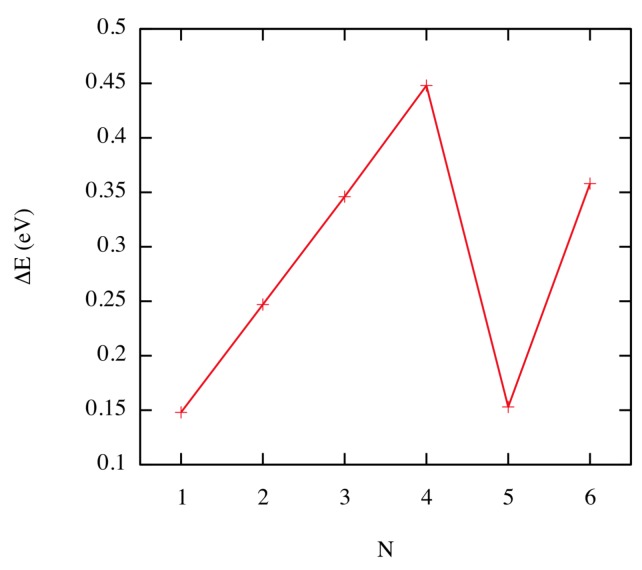
(Color online) Migration energies as a function of the He cluster size. Taken from Perez et al. [23].

The corresponding migration energy [23] as a function of cluster size is presented in Figure 5. Coinciding with the unusual structure of the clusters highlighted previously, the cluster with N=5 He atoms exhibits a very low diffusion barrier, indicating that its mobility is significantly higher.

**Figure 6:**
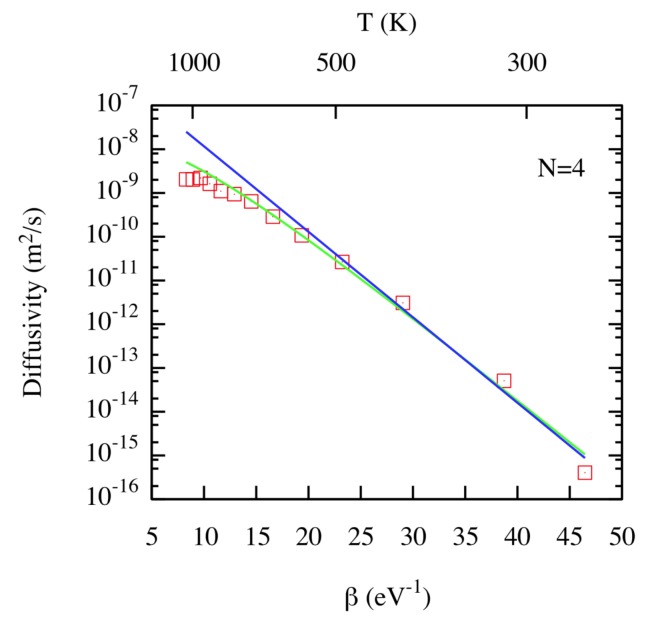
(Color online) Diffusivity as a function of $\beta =\frac{1}{k_{B}T}$ for a He cluster with size N=4. Notation: red squares: MD results; blue line: Harmonic Transition State Theory (HTST); green line: Superbasin (SB)-HTST. Taken from Perez et al. [23].

The diffusivity of the different clusters, as a function of temperature, was obtained from the time-dependent Mean Squared Displacement (MSD) via a linear fit. As an example, in Figure 6, we show the results corresponding to N=4 (similar behavior is seen for N=1,6). For the cluster sizes considered in this study, the main result was the departure from a standard Arrhenius behavior. Additional insight is gained by considering what Perez et al. [23] termed Superbasin Harmonic Transition State Theory (SB-HTST), from which a generalized Arrhenius expression is obtained with an activation energy that becomes a function of temperature. In very good agreement with the MD results, SB-HTST predicts the downward bending of the slope of the Arrhenius curve as the temperature rises, due to the increase in the sampling frequency of higher-lying energy basins.

**Figure 7:**
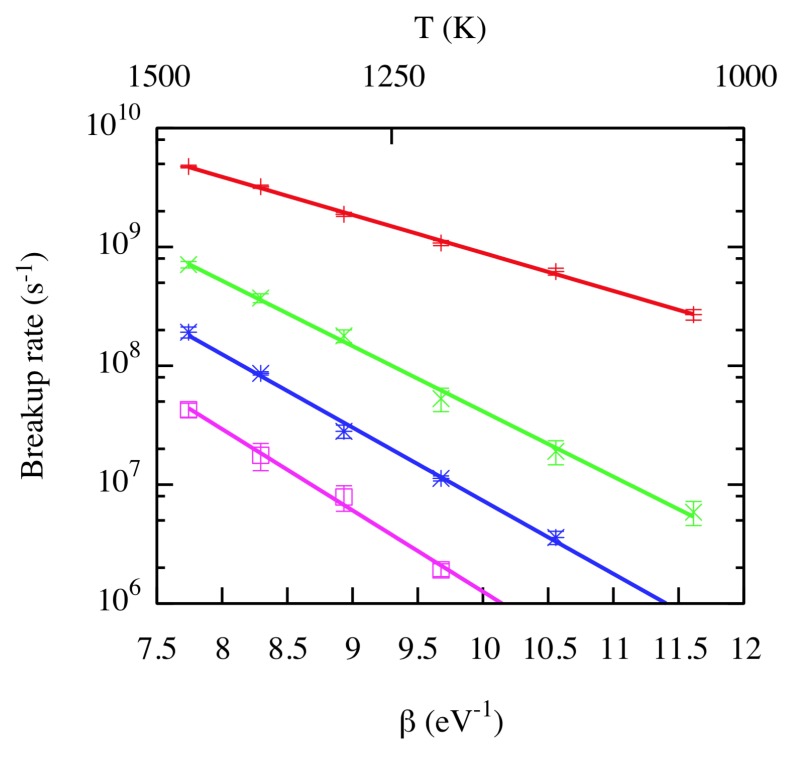
(Color online) Breakup rate as a function of β. Red +: N=2; green ×: N=3; blue *: N=4; pink ☐: N=5. Lines correspond to Arrhenius fits. Taken from Perez et al. [23].

On the other hand, even if the formation of He clusters is energetically favorable, configurational entropy effects favor isolated He atoms at low concentrations and high temperatures, which motivates the consideration of breakup reactions. After a careful definition of bound and unbound radii, Perez et al. [23] directly determined the breakup rate of He clusters in a range of temperatures between 1000 K and 1500 K using MD. Their results indicated a fast decrease of the breakup rate (and an increase of activation barriers) as the He cluster size increases, which is shown in Figure 7, in agreement with the fact that the binding energy per He atom increases with the cluster size.

**Figure 8:**
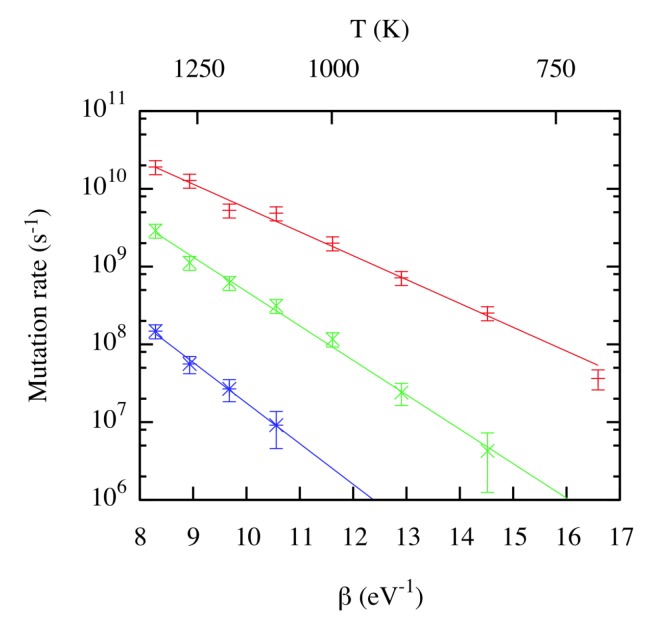
(Color online) Mutation rate as a function of β. Red +: N=7; green ×: N=6; blue *: N=5. Lines corresponds to Arrhenius fits. Taken from Perez et al. [23].

Concerning the trap mutation process, Perez et al. [23] also obtained the mutation rates as a function of temperature, which are shown in Figure 8. The mutation rate increases sharply with size, with a clear Arrhenius behavior over the range of temperatures considered. Other MD calculations [24] also exhibit an Arrhenius behavior, although the values reported for the characteristic times were approximately two orders of magnitude higher. As expected, the larger the cluster, the higher the mutation rate. Further compounding this effect is the fact that mutation is reversible at smaller *N*, i.e., that the W vacancy and W interstitial can recombine, restoring the He atoms to interstitial positions.

The results presented in this section provide a comprehensive characterization of the kinetics of small interstitial He clusters in bulk W. The behavior close to surfaces has been also studied via MD simulations [27,28] and DFT calculations [29]. This detailed analysis of He cluster diffusion, breakup, and mutation into He bubbles can feed, e.g., cluster dynamics [30,31] or kinetic Monte Carlo [32,33] models able to describe the performance of W at the mesoscale. As an example, by recording the cluster pressure and using an object kinetic Monte Carlo code, Valles et al. [33] showed that the elastic strain energy in the interior of the grains of nanocrystalline W was significantly lower than the one calculated for monocrystalline W, indicating that nanocrystalline W has a better mechanical response under He irradiation.

### 3.2. Mobility of Small Vacancy/Helium Complexes

A further step in understanding the He damage in W is the study of the kinetics of small vacancy/helium ($V_{N}{\mathrm{He}}_{M}$) complexes. After implantation, He atoms diffuse and form clusters, as discussed in the previous section. Eventually, these He clusters are capable of self-trapping via the nucleation of a Frenkel pair (W interstitial/vacancy recombination is prevented by He atoms filling the vacancy) or of binding to preexisting W vacancies. Perez et al. [34] performed AMD simulations via the Parallel trajectory Splicing (ParSplice) method to study the motion of the complexes involving one or two W vacancies, characterizing their diffusivity.

**Figure 9:**
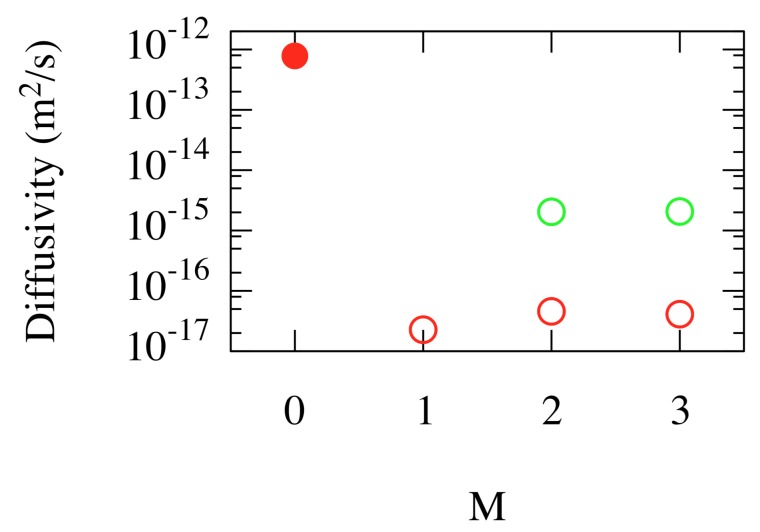
(Color online) Diffusivity of low He content $V_{N}{\mathrm{He}}_{M}$ complexes at T=1000 K. *N* and *M* denote the number of W vacancies and He atoms, respectively. Red: N=1; green: N=2. The filled circle symbol corresponds to a single W vacancy without He atoms. Open symbols are upper bounds at a 90% confidence level for cases where the complexes did not diffuse on accessible simulation timescales. Taken from Perez et al. [34].

A first striking observation is the effect of a single He atom on the mobility of W vacancies. Perez et al. [34] found that adding a He atom to a single W vacancy (N=1) causes the diffusivity to change by at least four orders of magnitude, from ∼$10^{-12}\;\;m^{2}/s$ to a value below ∼$10^{-16}\;\;m^{2}/s$ (with 90% confidence). Note that previous DFT calculations [35] provided explicit values for the change in migration energy (from 1.81 eV–4.83 eV), which explains the immobilization of the $V_{1}{\mathrm{He}}_{1}$ complex on ms timescales. Similar values were obtained for two and three He atoms, as shown in Figure 9.

A di-vacancy (N=2), on the other hand, is weakly bounded. Over long times, the dimer breaks apart. In this case, the single He atom immobilizes just one vacancy. However, over longer time scales, the dimer may reform, eventually allowing the He atom to jump to the other vacancy. An additional He atom, as seen in Figure 9, immobilizes the dimer over tens of μs time scales.

An increase of the *M*-to-*N* ratio leads to He bubble growth via Frenkel pair nucleation [36,37]. Perez et al. [34] highlighted that the Frenkel pair nucleation process is reversible. Using ParSplice simulations, Perez et al. [34] found that the rates at which nucleation and annihilation (untrapping) occur are quite sensitive to He content: as *M* increases, the nucleation rate increases, while the annihilation rate decreases, as He pressure favors nucleation, but counteracts annihilation. Similar results for He in Fe have been obtained by Gao et al. [38] also using an AMD approach.

**Figure 10:**
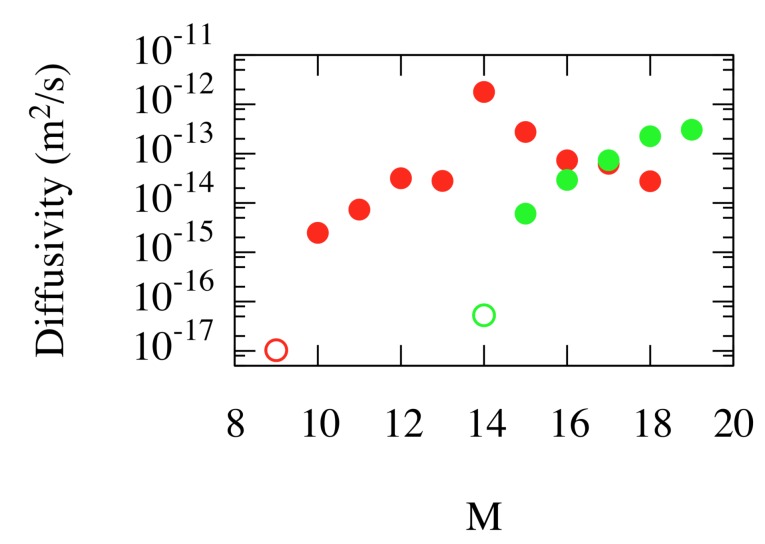
(Color online) Diffusivity of high He content $V_{N}{\mathrm{He}}_{M}$ complexes at T=1000 K as estimated from kinetic Monte Carlo simulations informed by ParSplice simulations. Red: N=1; green: N=2. Filled symbols correspond to estimates of the diffusivity, while open symbols are upper bounds at a 90% confidence level for cases where the complexes did not diffuse on accessible simulation timescales. Taken from Perez et al. [34].

Annihilation is not restricted to the reverse process of the last nucleation event, but instead, many variants are possible due to the diffusion of W interstitial around the vacancies. Sequences of such nucleation/interstitial migration/annihilation events give rise to net migration of the cluster. Figure 10 shows that the diffusivity of complexes depends sensitively on the He content, through the dependence of the nucleation and annihilation rates.

**Figure 11:**
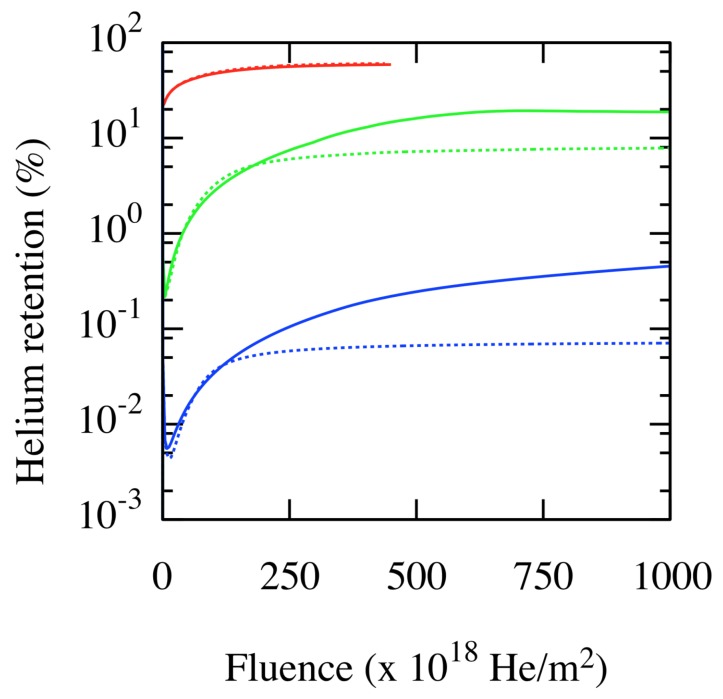
(Color online) He retention as a function of fluence. Red: $4\times 10^{25}\;\;\mathrm{He}/m^{2}/s$; green: $4\times 10^{23}\;\;\mathrm{He}/m^{2}/s$; blue: $4\times 10^{22}\;\;\mathrm{He}/m^{2}/s$. Continuous and dashed lines correspond to immobile and mobile complexes, respectively. Taken from Perez et al. [34].

In order to evaluate the importance of considering the mobility of such complexes, Perez et al. [34] performed mesoscale cluster dynamics simulations using the Xolotl model of microstructural evolution [30]. Figure 11 shows the overall retention of He atoms within the W wall when the diffusion of He/vacancy complexes is either allowed or forbidden. The results clearly showed that the mobility of these complexes provides an efficient outgassing pathway for He. These results also demonstrate that incorrect conclusions can be reached from standard MD simulations, as their limited time-scale restricts the set of events that can be observed. Surface effects on the stability of helium-vacancy complexes, on the other hand, have been recently studied via DFT calculations [39].

## 4. Nucleation and Growth of He Bubbles

In this section, we focus on the growth of He bubbles after self-trapping, whereby additional He atoms are captured by helium/vacancy complexes, driving the nucleation of additional Frenkel pairs and the formation of dislocation lines around the bubbles.

Sandoval et al. [37] studied the growth of isolated He bubbles using AMD simulations in order to isolate the effect of the growth rate on the microstructural evolution. Starting with a W vacancy filled with eight He atoms, located 1.9 nm below the surface of the material, Sandoval et al. [37] carefully inserted He atoms at constant time intervals (the diffusion process of He atoms and their capture by the bubble were not explicitly considered). The growth rates spanned six orders of magnitude, from $10^{12}$–$2\times 10^{6}$ $\mathrm{He}\;s^{-1}$, which can be associated with He fluxes in the range of $10^{30}$–$10^{24}$ $\mathrm{He}\;m^{-2}s^{-1}$, the last one being on the order of magnitude of fluxes expected at ITER [40].

**Figure 12:**
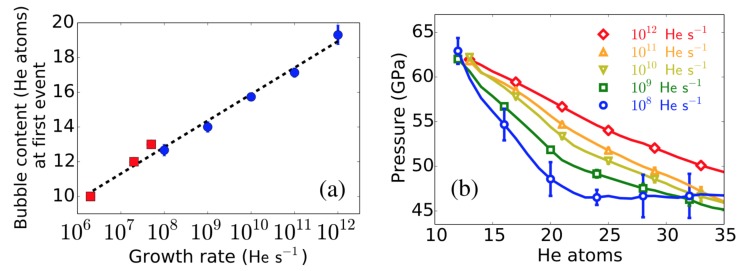
(Color online). Bubble growth as a function of the He insertion rate. (**a**) Average content of He atoms in the bubble at the time of the first detected event. Points with no error bars (red squares) are obtained from a single Parallel Replica Dynamics (ParRep) simulation. Points corresponding to rates ≥$10^{11}$ $\mathrm{He}\;s^{-1}$ were obtained via standard MD simulations. (**b**) Average pressure in the He bubble as a function of the He content and growth rate. Taken from Sandoval et al. [37].

Successive incorporation of He atoms in a bubble increases its pressure, which is the driving force for nucleation of Frenkel pairs, as has been shown by standard MD simulations [36]. The nucleation of Frenkel pairs increases the bubble size and partially releases the pressure in the bubble. Prismatic 〈111〉 dislocation loops, formed by the aggregation of W interstitials, are emitted from the bubble. Sandoval et al. [37] observed clear kinetic differences as a function of the growth rate. For instance, the first detected event (the nucleation of a Frenkel pair) required fewer He atoms at slower growth rates, as shown in Figure 12a.

After nucleation of a Frenkel pair, the new W vacancy increases the bubble volume, while the W interstitial becomes a 〈111〉 crowdion tangent to the bubble. If the growth rate is slow enough, the crowdion is able to move around the bubble. Simulations show that subsequent interstitial emissions (driven by the constant addition of He) are most likely to occur in the neighborhood of interstitials already decorating the bubble, hence forming an incipient 〈111〉 dislocation line. For shallow bubbles, these dislocations elastically interact with the W surface, which drives their motion to the side of the bubble closer to the surface. This in turn favors subsequent interstitial emission from the surface-facing side of the bubble, which causes a preferential growth towards the surface. The dislocation arc eventually detaches from the bubble forming a 〈111〉 dislocation loop, which glides to the surface displacing W atoms and increasing the surface roughness. Note that, in general, as the growth rate is lowered, the probability of nucleating a new Frenkel pair before the next insertion increases, leading to lower pressure because of smaller helium/vacancy ratios, as seen in Figure 12b. This allows the definition of slow and fast growth regimes based on the speed at which W interstitials (crowdions) can diffuse around the bubble. At fast growth rates, typical of the ones commonly used in standard MD simulations, the emission of W interstitials is fast compared to their diffusion around the bubble. Therefore, dislocation lines grow where they are first nucleated, generating a more isotropic growth, delaying the bursting point as compared to the slow growth regime where emitted interstitials have time to diffuse around the bubble, facilitating the interaction with the surface and leading to a more directional growth process.

**Figure 13:**
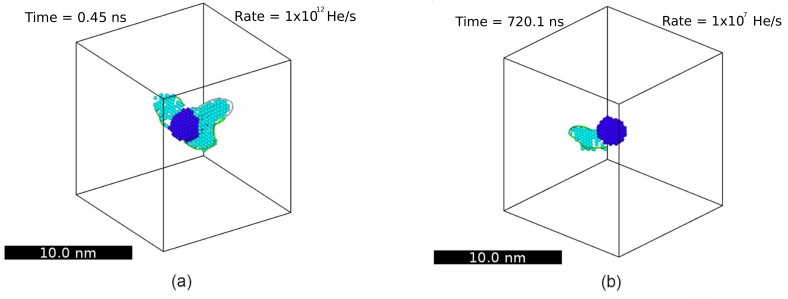
(Color online) Representative snapshots highlighting the dislocation loops attached to a growing He bubble for (**a**) fast and (**b**) slow growth rates. Dark and light spheres correspond to W vacancies and W interstitials, respectively. The detection of point defects is performed by the Wigner–Seitz defect analysis tool implemented in OVITO [42]. Taken from Sandoval et al. [41].

The growth of deeper and bigger He bubbles was studied by Sandoval et al. [41], following the approach described previously, where MD and AMD simulations were performed to explore the role of the growth rate, whose values spanned six orders of magnitude ($10^{6}$–$10^{12}$ $H\;s^{-1}$). In this case, the bubble was created 6 nm below the surfaces. As observed in Sandoval et al. [37] for shallow bubbles, fast growth rates lead to higher pressures than slow growth rates. Between successive insertions, at slow growth rates, the system is able to explore its phase-space more thoroughly, eventually activating relaxation mechanisms such as the nucleation of Frenkel pairs, which allows for the release of the pressure. In addition, a remarkable difference is observed between simulations with different growth rates, corresponding to the number of coexisting dislocation lines attached to the bubble. As is shown in Figure 13, multiple dislocations were observed at fast growth rates, in contrast to only one at slower rates. This was again attributed to the insufficient time for the interstitials to reorganize around the bubble when the interstitial emission rate was fast compared to their diffusion rate.

Using a Parallel Replica Dynamics (ParRep) [43] simulation, Sandoval et al. [41] showed that the nature of the dislocation structure around a growing bubble strongly depends on the growth rate. Specifically, they took, as the initial ParRep configuration, a dislocation structure composed of many arcs obtained from an MD simulation of a bubble growing at a fast rate. The entangled dislocation lines evolved to a single dislocation via a reorganization of W interstitials, which was then released from the bubble as a single loop.

Recent works have advanced this knowledge by studying the loop-punching mechanism for large bubbles at low temperatures (300 K) [44], the energetics and kinetics of He bubble growth for insertion rates in the fast growth regime (∼30 ps between He insertions) [45], the lifetimes of non-growing He bubbles close to W surfaces [46], and the effect of strain fields [47]. MD and AMD results describing the bubble bursting process have also been incorporated into the cluster dynamics model Xolotl [31].

**Figure 14:**
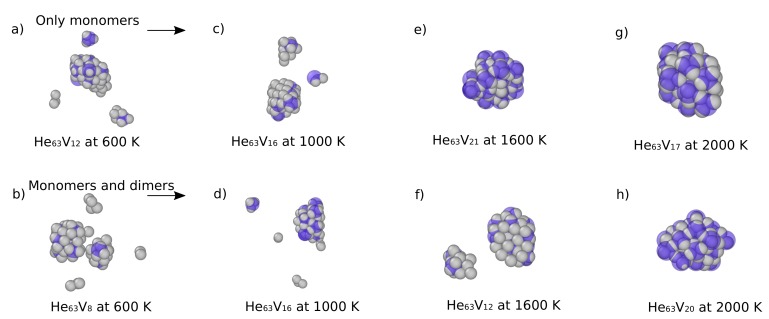
(Color online) Formation of He bubble networks. The top row shows snapshots after 55 He monomers have been inserted at a rate of $1\times 10^{9}$ $\mathrm{He}\;s^{-1}$. The bottom row shows snapshots after insertion of 49 He monomers, at the same rate, plus three He dimers inserted at t= 10, 30, and 50 ns. Gray and blue spheres denote He atoms and W vacancies, respectively. Taken from Sandoval et al. [48].

Including the diffusion process of He clusters to study the growth process of He bubbles adds interesting aspects to the simulation results, as shown by Sandoval et al. [48]. He monomers and dimers were introduced in a simulation box thermalized at different temperatures in the range [600, 2000] K. After nucleation of an initial He bubble and a W interstitial (crowdion) via self-trapping, incoming He clusters were allowed to diffuse freely and interact with them. The simulations showed that incoming He atoms strongly interact with the crowdion structure developed around a bubble, either impeding their incorporation into the bubble or even trapping them. If an He atom remains trapped in the crowdion structure for a sufficiently long time, it can eventually be joined by a newly-inserted He atom, forming a dimer. Sandoval et al. [48] also showed that the interaction with a He dimer is stronger, as compared to single He atoms. The simulations revealed that if the He-dimer, already trapped in the crowdion structure, captured two additional He atoms, the nucleation of a new He bubble via self-trapping was highly probable. Subsequently, as new bubbles grow, more crowdions are generated, which, in turn, can capture additional He atoms. A network of He (nano-)bubbles is formed, which is strongly dependent on the temperature, as shown in Figure 14. High temperatures reduce the probability that He atoms would remain trapped by the crowdions long enough to facilitate the nucleation of new He bubbles. Furthermore, at low He fluxes the trapped clusters would likely have time to de-trap from the crowdion structures, which affects the formation of the network.

## 5. Interaction between He-Clusters and He-Bubbles

**Figure 15:**
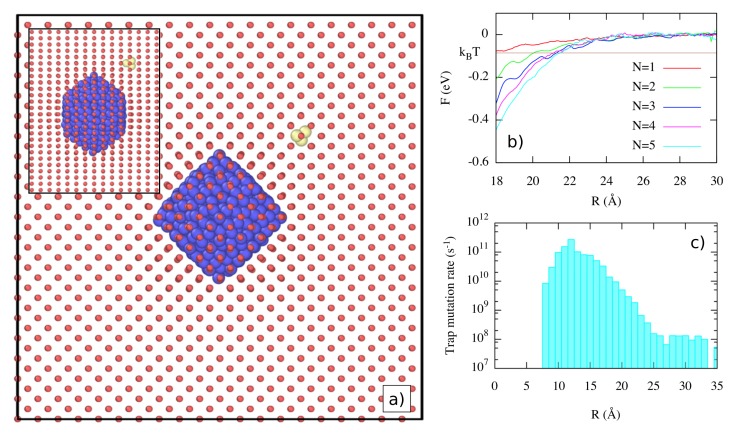
(Color online) Interaction of He clusters with He bubbles. (**a**) Simulation setup: W, substitutional He, and interstitial He atoms are shown as red, blue, and yellow spheres, respectively. The plot axes are aligned with [100] directions. An He cluster containing three atoms is placed in [110] orientation relative to the center of the bubble. The inset corresponds to a view from a [110] direction. (**b**) Free energy of He${}_{N}$ clusters as a function of the distance to the center of the bubble. (**c**) Radially-averaged trap mutation rates as a function of the distance to the center of the bubble for N=5. Taken from Perez et al. [49].

Now, we extend the observations considered in the previous section to include the interaction between pre-existing bubbles and He clusters. Perez et al. [49] characterized the thermodynamics and kinetics of these interactions, showing that, in addition to attracting clusters, He bubbles also enhance the probability of trap-mutation in their neighborhood. In Figure 15a we show the simulation setup used by Perez et al. [49]. In a cubic simulation box containing 16,000 W atoms, a rhombic dodecahedral bubble with (110) facets was constructed by creating a void of 175 W vacancies, which was subsequently filled with 481 He atoms (in the original paper [49], the bubble geometry used was incorrectly described). The system was equilibrated at a temperature of T=1000 K. Previous MD simulations indicated that the internal pressure was 26 GPa, a typical value for a growing bubble that has just released a dislocation loop. In addition to the large bubble, individual ${\mathrm{He}}_{N}$ (*N* = 1–6) clusters are introduced in the simulation cell.

Using a direct histogram method, Perez et al. [49] obtained the free energy of He clusters at different positions around the bubble. The free energy gradually decreases at short range, as seen in Figure 15b, while the elastic interaction becomes stronger as the He cluster size increases. The capture distance goes from ∼18 Å for N=1–∼22 Å for N=6. Furthermore, the He cluster–bubble interaction presents a notable angular dependence because of the elastic anisotropy. He clusters are channeled along [100] directions, as approaches along [111] directions are strongly disfavored.

Although the incorporation of He clusters into existing bubbles is thermodynamically favored, there also are mechanisms that might obstruct the bubble growth. One of them is trap-mutation, where the cluster becomes a satellite nanobubble. Perez et al. [49] computed the trap mutation rates for He${}_{N}$ clusters as a function of the position around the bubble. Their results clearly showed an enhancement of the trap mutation rate for distances going from 8 Å to 25 Å. Figure 15c shows the case corresponding to N=5, where the asymptotic behavior to bulk values at long distances is clear, while at short distance, the trap-mutation rate increases by almost three orders of magnitude. Much like the interaction with the dislocations above, strains due to the surrounding microstructure can enhance the probability of self-trapping.

Using AMD simulations, Perez et al. [49] determined the role of the satellite nanobubbles in the bubble growth process. One interaction mechanism observed corresponds to the annihilation of the satellites, for N=1 or 2 and short distances, by means of a W interstitial emitted by the bubble via a Frenkel pair nucleation. The nanobubble becomes a mobile interstitial cluster again, which is easily absorbed by the bubble. At longer distances, the annihilation of W vacancies in satellite bubbles can be activated when 〈111〉 dislocation loops emitted by the bubble impinge upon the nanobubble; this mechanism is more significant for small *N*. For larger values of *N*, we observed that transfer of He atoms to the bubble preceded annihilation. In particular, it was observed that, for N∈(4,5,6), dislocation loops and nanobubbles were transiently bound, which facilitated the transfer of He to the bubble.

## 6. Interaction between He-Clusters/Bubbles and Defects

We have so far considered the nucleation and growth of He bubbles in initially pristine crystal lattices or in lattices with a controlled configuration of vacancies where He atoms have been carefully introduced. All crystal defects (vacancies, interstitials, and dislocations) were generated in the process of nucleation and growth. In this section, we highlight some recent results obtained via AMD simulations, which consider pre-existing defects, in particular Grain Boundaries (GBs).

**Figure 16:**
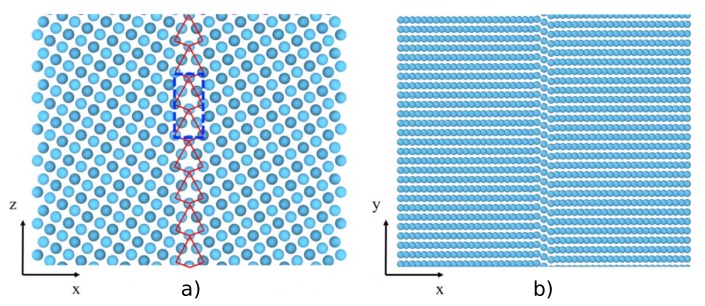
(Color online) Structure of the Σ5[100](310) tilt grain boundary in W. (**a**) Along the tilt axis. (**b**) along $\left[130\right]{}_{\mathrm{grain}\;1}$/$\left[310\right]{}_{\mathrm{grain}\;2}$. Taken from Liu et al. [50].

Recently, Liu et al. [50] studied the process of He-bubble nucleation and growth in the neighborhood of a Σ5[100](310) tilt GB. This GB possesses a well-ordered structure, stable at temperatures typical of a fusion environment (1000 K in that work), as shown in Figure 16.

**Figure 17:**
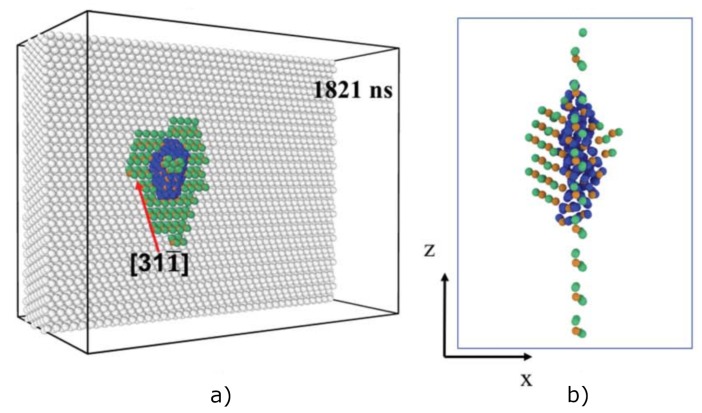
(Color online) Final atom configuration of a ParRep simulation of the He growth process of a bubble pre-nucleated at the grain boundary. (**a**) Perspective view showing the boundary of one of the grains. (**b**) Side view showing only the defects. White, orange, green, and blue spheres denote non-defective W atoms, W vacancies, W interstitials, and He atoms, respectively. Taken from Liu et al. [50].

Liu et al. [50] used a simulation supercell for ParRep simulations initially containing 39,200 atoms, with periodic boundary conditions applied along the *y* and *z* directions. Similar to Sandoval et al. [37], eight He atoms were placed inside a pre-existing vacancy at the center of the GB plane. New He atoms were inserted inside the bubble at a rate of 1 He atom/10 ns, a value that corresponds to the slow growth regime in bulk conditions [37], close to what is expected at ITER for surface GBs [40]. During growth, W interstitials were emitted within the GB, and some of them escaped away from the nanobubble. As the bubble grew, it was observed that self-interstitial-atom loops were not formed, contrary to the behavior in the bulk, due to the strong binding of W interstitials to the GB plane. Eventually, a halo of W interstitials was formed around the bubble, trapped at the GB, as shown in Figure 17, corresponding to the final snapshot of the ParRep simulation.

**Figure 18:**
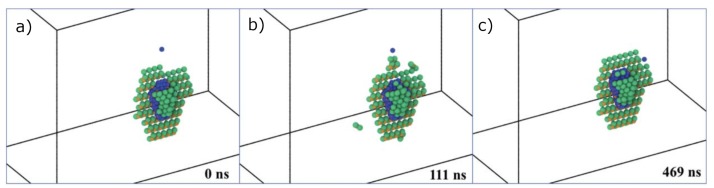
(Color online) Snapshots of the temporal evolution of the interaction of an excess He atom and a He bubble, both located at the grain boundary, obtained via ParRep simulations. (**a**) 0 ns, (**b**) 111 ns, and (**c**) 469 ns. White, orange, green, and blue spheres denote non-defective W atoms, W vacancies, W interstitials, and He atoms, respectively. Taken from Liu et al. [50].

In a second set of simulations, Liu et al. [50] evaluated the effect of the W interstitial halo on the arrival of a diffusing He atom. Snapshots corresponding to these simulations are shown in Figure 18. It was observed that the W halo attracts the incoming He atom, but prevents it from reaching the bubble, which suggests that the W halo may limit the diffusion of He atoms into the bubble, affecting the bubble growth process. This hypothesis was further supported by directly computing the diffusion barriers for He using climbing-image nudged elastic band calculations [25,51].

In a last set of ParRep simulations, Liu et al. [50] introduced He atoms randomly into the GB at a rate of one He atom/100 ns. Inserting He atoms directly into the GB plane is supported by the fact that He atoms introduced into bulk W quickly migrate to the GB, as shown by recent simulations [27,30,52], where a sink segregation strength has been defined and calculated for some symmetric tilt grain boundaries, as well as for a few surface orientations. As in the bulk, He atoms migrating in the GB eventually encounter other He atoms, trap-mutating and nucleating bubbles. Again, contrary to the bulk case, interstitials emitted during the bubble growth process cannot escape, as previously described. As a result, for a given population of He atoms, a more evenly-dispersed set of He nanobubbles is expected at a GB, as compared to the bulk. After this saturation stage, new He atoms would arrive directly to the reconstructed GB region, re-initiating the bubble growth process. Further investigation is required to determine the bubble nucleation and growth process in other types of GBs to determine the generality of this bubble growth mode.

## 7. Discussion

The key defining characteristic of the AMD methods is their ability to extend the time scale of atomistic simulations to times further, in some cases much further, than conventional MD, while retaining near-full fidelity with the underlying atomistic interactions. This enables simulations that are simply impossible otherwise, probing behavior that is otherwise opaque. Many of the examples discussed in this overview exemplify this behavior, as we have elucidated atomic-scale mechanisms that would not be found if limited to MD time scales.

Just as importantly, other methods for accelerating time scales, such as Adaptive Kinetic Monte Carlo (AKMC) [53,54], would be challenging, if not impossible, to apply to some of the scenarios discussed here. In ParRep, the definition of a state can be made very generally, and this was particularly valuable when simulating bubbles, where the dynamics of the fast He atoms could be ignored. Methods such as AKMC and, to be clear, TAD, which require explicitly finding saddle points for all events, would not be applicable to systems with such fast moving degrees of freedom. Thus, while those methods are certainly powerful and provided new insight into some of the problems described here, there are limitations in using those methods in simulations involving gas bubbles.

For example, in scenarios in which the He gas is still in the W matrix, TAD proved effective at describing the kinetics of the resulting interstitial clusters. These TAD simulations revealed complex behavior versus cluster size that would be difficult to guess a priori. However, once gas bubbles nucleate, methods such as TAD would simply be impossible to apply. Using ParRep, where the states could be defined based on the W subsystem, the types of simulations can be extended significantly. In particular, ParRep enabled long-time simulations of bubbles in multiple environments with varying types of boundary conditions, providing new insight into how bubbles grow.

The most important consequence of extending the time scale of these simulations is that new mechanisms start to dominate. In most of the examples discussed here, there are multiple atomic-scale mechanisms with different rates that compete to drive the evolution of the material. If the time scale is not extended, the external drive applied to the system, in this case, the introduction of He, has to be so fast that other processes simply cannot occur on the time scale of the simulation. For example, if He is introduced too fast into a growing bubble, the emitted W interstitials are simply frozen in place, and the bubble grows qualitatively differently than if those interstitials can migrate and interact with the surrounding microstructure. Similarly, if the bubbles are grown more naturally, via the arrival of He from the matrix, the rate of He introduction changes whether the He reaches the central bubbles or not. In the case of the migration of He-V clusters, there is no external drive, but extending the time scale of the simulation allowed for both Frenkel pair formation and, critically, annihilation. If that annihilation had not been observed, neither would the net migration of the cluster. Thus, we see repeatedly that the observed behavior strongly depends on which mechanisms are active over the course of the simulation.

It should be noted that AMD methods are not a panacea for all simulations at the atomic scale. There are cases where AMD approaches will not provide any significant benefit over conventional MD. For example, the dynamics of the He within the bubbles cannot be accelerated as their dynamics are simply so fast. In the cases described here, this was not a critical limitation as the W dynamics dictate the system evolution. However, if all of the key behavior was related to the dynamics of the fast moving system, the AMD methods would provide only marginal benefit. As a general rule, what is already fast cannot be significantly accelerated.

Another limitation involves the system size. As the system size increases, so does the computational cost. Depending on the nature of the scaling, the AMD methods scale worse than MD, the worst situation being when the overall rate of events increases with system size. Thus, the simulations presented here are limited in how deep the bubbles are below the surface, a direct consequence of the need for larger system sizes and the issue with scaling. There are developments to overcome these limitations, and significant progress has been made; however, this is one of the ongoing challenges in applying these methods.

However, in spite of these limitations, the insights gained from these simulations extend our knowledge of how gases behave in metals, not only for fusion-relevant conditions, but more generally. They provide direct atomic-level information about key mechanisms that can be directly used in higher level models and have been shown to significantly change predictions at the meso-scale. In conjunction with conventional MD simulations, atomic scale simulations using AMD methods can provide high fidelity information about the atomistic mechanisms that drive material evolution in harsh conditions where kinetics are all-important.

## 8. Conclusions

We have reviewed some recent results concerning the modeling and simulation of helium effects in tungsten in the context of nuclear fusion power, with special focus on the application of accelerated molecular dynamics methods. We have considered the implantation process of helium atoms in the tungsten matrix, the subsequent diffusion of helium clusters, the process of nucleation and growth of helium bubbles, the interaction between helium clusters and bubbles, and the role of tungsten defects (interstitials, dislocations, and grain boundaries). A manifest outcome of this review corresponds to the importance of taking into account an extended range of time scales in order to include the dominant mechanisms determining the evolution of the surface microstructure in tungsten as a plasma-facing material.
